# Comprehensive molecular characterization of *Methylobacterium extorquens* AM1 adapted for 1-butanol tolerance

**DOI:** 10.1186/s13068-016-0497-y

**Published:** 2016-04-11

**Authors:** Bo Hu, Yi-Ming Yang, David A. C. Beck, Qian-Wen Wang, Wen-Jing Chen, Jing Yang, Mary E. Lidstrom, Song Yang

**Affiliations:** School of Life Science, Shandong Province Key Laboratory of Applied Mycology, and Qingdao International Center on Microbes Utilizing Biogas, Qingdao Agricultural University, Qingdao, Shandong Province China; Department of Chemical Engineering, University of Washington, Seattle, WA USA; eScience Institute, University of Washington, Seattle, WA USA; Central Laboratory, Qingdao Agricultural University, Qingdao, Shandong Province China; Department of Microbiology, University of Washington, Seattle, WA 98195-1750 USA; Key Laboratory of Systems Bioengineering, Ministry of Education, Tianjin University, Tianjin, China; Industrial Product Division, Intrexon Corporation, South San Francisco, CA 94080 USA

**Keywords:** *Methylobacterium extorquens* AM1, 1-Butanol tolerance, Adaptive evolution, Whole genome sequencing, Global metabolome analysis, Carotenoid

## Abstract

**Background:**

The toxicity of alcohols is one of the major roadblocks of biological fermentation for biofuels production. *Methylobacterium extorquens* AM1, a facultative methylotrophic α-proteobacterium, has been engineered to generate 1-butanol from cheap carbon feedstocks through a synthetic metabolic pathway. However, *M. extorquens* AM1 is vulnerable to solvent stress, which impedes further development for 1-butanol production. Only a few studies have reported the general stress response of *M. extorquens* AM1 to solvent stress. Therefore, it is highly desirable to obtain a strain with ameliorated 1-butanol tolerance and elucidate the molecular mechanism of 1-butnaol tolerance in *M. extorquens* AM1 for future strain improvement.

**Results:**

In this work, adaptive laboratory evolution was used as a tool to isolate mutants with 1-butanol tolerance up to 0.5 %. The evolved strains, BHBT3 and BHBT5, demonstrated increased growth rates and higher survival rates with the existence of 1-butanol. Whole genome sequencing revealed a SNP mutation at *kefB* in BHBT5, which was confirmed to be responsible for increasing 1-butanol tolerance through an allelic exchange experiment. Global metabolomic analysis further discovered that the pools of multiple key metabolites, including fatty acids, amino acids, and disaccharides, were increased in BHBT5 in response to 1-butanol stress. Additionally, the carotenoid synthesis pathway was significantly down-regulated in BHBT5.

**Conclusions:**

We successfully screened mutants resistant to 1-butanol and provided insights into the molecular mechanism of 1-butanol tolerance in *M. extorquens* AM1. This research will be useful for uncovering the mechanism of cellular response of *M. extorquens* AM1 to solvent stress, and will provide the genetic blueprint for the rational design of a strain of *M. extorquens* AM1 with increased 1-butanol tolerance in the future.

**Electronic supplementary material:**

The online version of this article (doi:10.1186/s13068-016-0497-y) contains supplementary material, which is available to authorized users.

## Background

The abundance of single carbon compounds such as methane and methanol represents novel opportunities for development of future alternative carbon feedstocks that are economically competitive with petrochemical synthesis yet at the same resource non-competitive with world demand for agricultural products [1, 2]. Methylotrophic bacteria are a group of widespread microorganisms that utilize single carbon compounds as the carbon and energy source, which could serve as environmentally friendly catalysts to generate chemicals and materials [3]. Among methylotrophs, *Methylobacterium extorquens* AM1 is the most well-understood microorganism with a history of biotechnological application such as the biosynthesis of amino acids and single-cell protein [4]. In the recent few years, elucidation of pathways involved in C1 and C2 metabolism and development of a next generation genetic tool set enable the direction of carbon flux in *M. extorquens* AM1 from methanol assimilation to the synthesis of higher value added products such as 1-butanol, a second generation biofuel [5–7].

However, the vulnerability of *M. extorquens* AM1 to solvent stress impedes its further development as a biofuel-producing platform. Preliminary experiments show that the growth of *M. extorquens* AM1 was inhibited in medium with 0.15 % (v/v) 1-butanol and completely stopped when the level of 1-butanol exceeds 0.25 % (unpublished data). Improvement of solvent tolerance through genetic manipulation requires substantial knowledge on molecular mechanisms of cell response to solvent stress, which could be complicated as revealed in other microorganisms [8–10]. So far only a few studies have reported the stress response of methylotrophs, such as the transcriptional analysis of *M. extorquens* AM1 to starvation and the effect of the PhyR regulon on the salt and ethanol tolerance of *M. extorquens* AM1 [11]. Therefore, it is difficult to improve 1-butanol tolerance of *M. extorquens* AM1 through manipulating a single gene or a few genes as a cluster.

Adaptive laboratory evolution (ALE) is a classic method to improve robustness of microbes to solvent, which artificially evolves microorganisms under solvent challenge for prolonged periods of time [12]. Combined with system biology approaches such as whole genome sequencing, transcriptional analysis, and metabolite profiling, the phenotype-genotype correlations can be established to determine the genetic basis of evolution, which can be used as a blueprint for rational design of industrial strains with desired traits [13, 14]. In a recent study towards increasing 1-butanol tolerance in *Escherichiacoli*, a large increase in solvent tolerance was rapidly achieved by combining laboratory evolution and genome shuffling of the evolved clones [15]. Atsumi et al. also reported an isobutanol-tolerant mutant isolated from serial transfers and identified primary mutations responsible for the increased isobutanol tolerance [16]. Evolved microorganisms demonstrated a variety of responses to solvent tolerance. Some bacteria are able to change the saturated-to-unsaturated fatty acids ratios or increase the length of the acyl-chains to solve the increased membrane fluidity caused by solvents [17]. *E. coli* could boost the synthesis of glucosamine-6-phosphate a precursor of peptidoglycan and lipopolysaccharide, to strengthen the cell wall as a barrier against isobutanol stress [16]. And many bacteria produce diverse metabolites to cope with the protein misfolding and instability in the presence of the solvent [18].

**Fig. 1:**
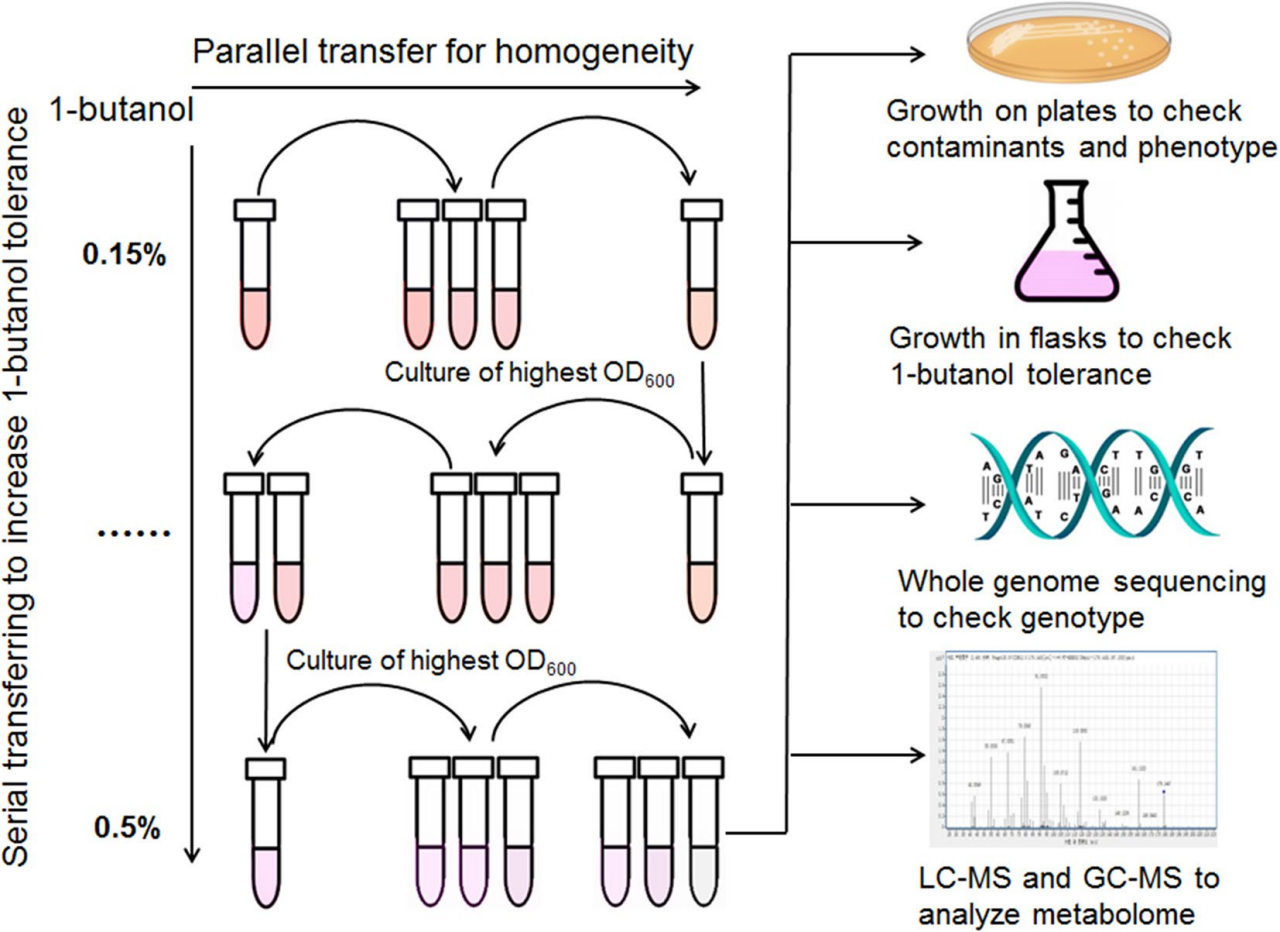
Experimental evolution of 1-butanol tolerance in *M. extorquens* AM1

In this work, as shown in Fig. 1, we applied an ALE method to isolate mutated strains of *M. extorquens* AM1 with increased tolerance to1-butanol. To understand the molecular basis of 1-butanol tolerance, we sequenced the whole genome of the evolved strain and aligned the reads with the published genome sequence. Moreover, global metabolomic analysis was carried out to discover metabolite changes between the 1-butanol-tolerant strain and wild-type. This research not only tests the approach of using ALE for strain improvement of *M. extorquens* AM1, but also provides valuable information to elucidate the genetic basis of solvent tolerance in *M. extorquens* AM1. This information opens the door for rational design of a 1-butanol-tolerant AM1 strain in the future.

## Results

### Isolation of 1-butanol-tolerant mutants of *M. extorquens* AM1

**Fig. 2:**
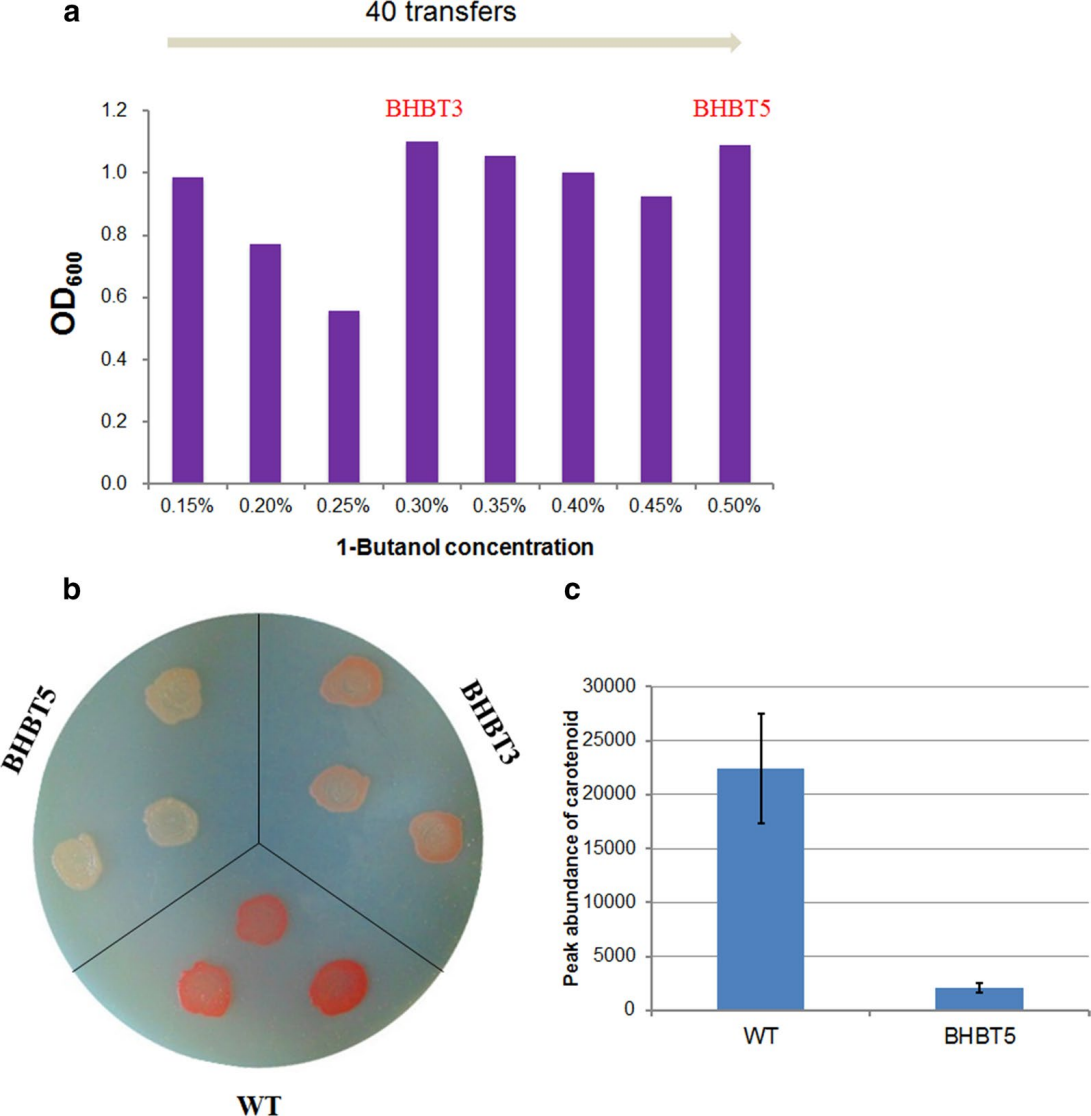
Butanol-tolerant strains of *M. extorquens* AM1 were obtained via serial transfer. **a** Final OD_600_ of *M. extorquens* AM1 from serial transfer experiments; **b** BHBT3 and BHBT5 produce less pink pigments than wild-type; **c** carotenoids abundance in BHBT5 and wild-type based on area under the peak attributed to carotenoids

A rifamycin-resistant derivative of *M. extorquens* AM1 (wild-type) was used as the parent strain for the evolution experiments [19, 20]. The wild-type strain was initially inoculated in hypho methanol medium containing 0.15 % (v/v) 1-butanol. After every 3–6 sequential transfers at the same concentration of 1-butanol, cultures of highest cell density were transferred into fresh medium in which the 1-butanol concentration was increased by 0.05 %. The OD_600_ of cultures transferred to medium with increased 1-butanol concentration is summarized in Fig. 2a. The end point cell density decreased with increasing 1-butanol concentration during the first three transfers. However, at 0.3 % 1-butanol, a portion of the cultures were able to grow to similar OD_600_ of that at 0.15 % 1-butanol, suggesting that critical genetic mutations related to 1-butanol tolerance may occur in these cultures. A single colony was separated from this culture and denoted as BHBT3, which was used for the following enrichment experiments. After a total number of 40 transfers, a mutant that was able to grow at 0.5 % 1-butanol was isolated and denoted as BHBT5. The sizes of BHBT3 and BHBT5 colonies on agar plates were identical with that of wild-type, but appeared to be white instead of pink (Fig. 2b). HPLC analysis showed that BHBT5 had much lower peak area of carotenoids compared to wild-type (Fig. 2c).

### Influence of 1-butanol on growth of *M. extorquens* AM1

**Fig. 3:**
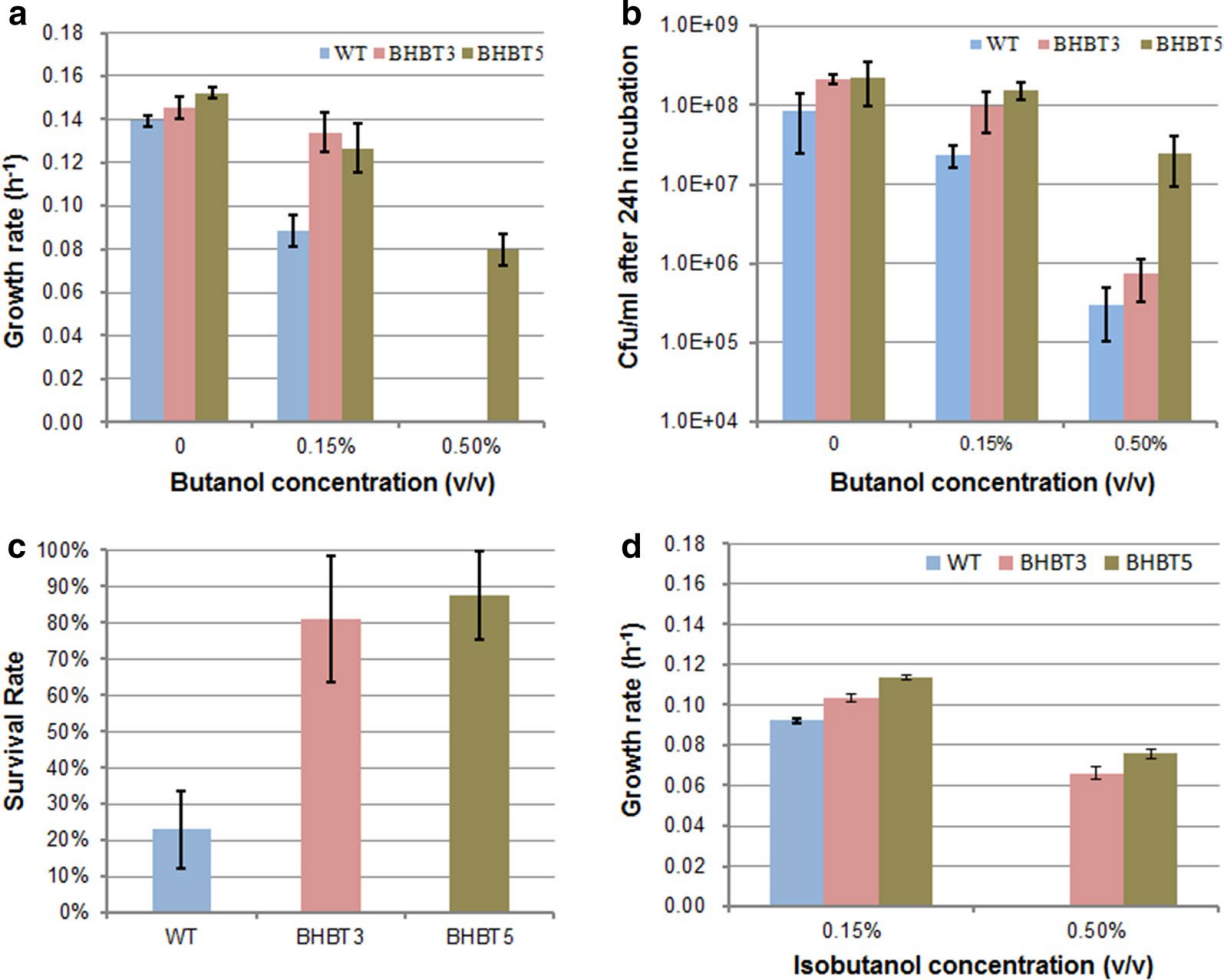
The evolved strains are more robust to high concentration of 1-butanol. **a** Comparison of growth rate between the WT and tolerant strains in the presence of butanol; **b** and **c** colony forming unit (cfu) and cell survival rate. The rate was calculated as cfu per ml of 5 % (v/v) butanol-exposed culture divided by that of control culture; **d** BHBT3 and BHBT5 strains displayed increased tolerance towards isobutanol. The data were presented as the mean plus STDEV calculated from triplicate biological replicates

To study 1-butanol tolerance of the BHBT3 and BHBT5 strains, growth of the wild-type, BHBT3, and BHBT5 strains in hypho methanol medium with different amounts of 1-butanol was investigated. No significant disparities were observed between growth rates of the three strains in medium without the addition of 1-butanol (Fig. 3a). However, the BHBT3 and BHBT5 strains were more tolerant to 0.15 % 1-butanol than the wild-type strain despite growth inhibition of all three strains (Fig. 3a). The growth rate of the wild-type strain was reduced by 36.5 % with the addition of 0.15 % 1-butanol while the growth rates of the BHBT3 and BHBT5 strains were only reduced by 7.5 and 16.9 % (Fig. 3a). In the presence of 0.5 % 1-butanol, only the BHBT5 strain was able to grow. The growth rate was reduced (50 %), but the final OD_600_ was similar to that in hypho medium without 1-butanol (Fig. 3a). Both wild-type and BHBT3 strains did not grow after 72 h of incubation. Cell counting results were consistent with OD_600_ measurements (Fig. 3b). The cell counts of the BHBT3 and BHBT5 strains were four- and sevenfold higher than that of wild-type, respectively, after 24 h of incubation in the presence of 0.15 % 1-butanol, while there were only twofold more viable cells in the BHBT3 and BHBT5 cultures than the wild-type culture in the absence of 1-butanol. Despite the low OD_600_ of both wild-type and BHBT3 strains incubated in 0.5 % 1-butanol medium, the cell count of the BHBT3 strain was three times higher than that of the wild-type strain. The number of viable BHBT5 strain cells was 2 orders of magnitude higher than the wild-type and BHBT3 strains. The survival rate of *M. extorquens* AM1 strains under the stress of high levels of 1-butanol was investigated by exposing wild-type, BHBT3, and BHBT5 strain to 6.17 % (v/v) of 1-butanol for 30 min. The survival rate of both the BHBT3 and BHBT5 strains was above 80 %, almost four times higher than that of the wild-type strain (23 %, Fig. 3c). This result shows that the evolved strains are more robust to a high concentration of 1-butanol. In addition, the BHBT3 and BHBT5 strains also displayed increased tolerance towards isobutanol (Fig. 3d), which was consistent with other reports showing that the isobutanol stress response in other bacteria was qualitatively similar to that of 1-butanol with respect to transcriptional and metabolite levels [21].

### 1-butanol production of BHBT5

**Fig. 4:**
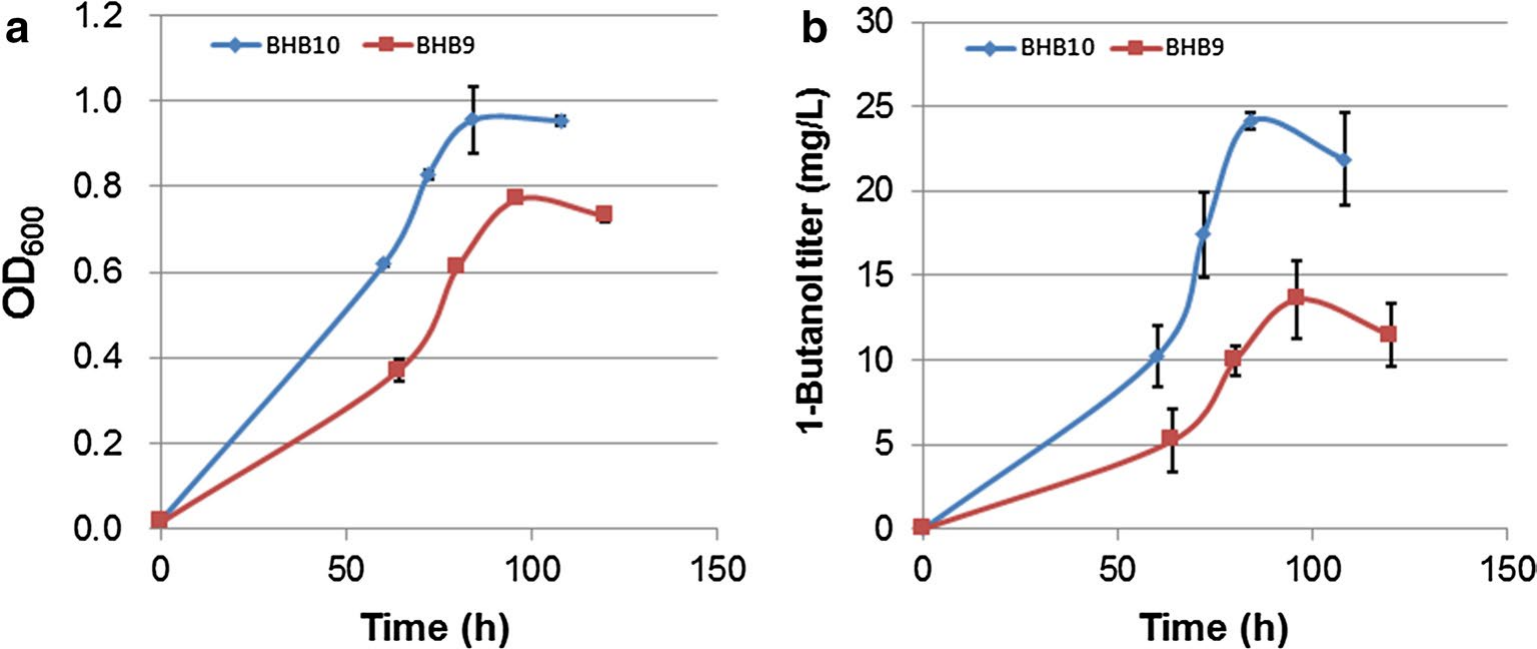
Butanol-tolerant strain harboring 1-butanol pathway demonstrated improved growth and 1-butanol production. Comparison of growth rate (**a**) and 1-butanol titer (**b**) between the tolerant strain (BHB10) and non-tolerant strain (BHB9)

To assess whether the genetic mutations would improve 1-butanol production, a plasmid harboring the 1-butanol synthetic pathway reported previously [6] was introduced into the BHBT5 strain by bacterial conjugation. 1-butanol production of the new strain, designated as BHB10, was investigated in hypho medium using ethylamine as the carbon source. Compared with the previous construct BHB9, both cell density and 1-butanol production of the BHB10 strain were increased (Fig. 4). After 72-h incubation, the BHB10 strain was able to produce a maximum of 25.5 mg/L 1-butanol, representing 87 % more than the original BHB9 strain. OD_600_-calibrated 1-butanol production of the BHB10 strain was also 30 % higher than that of the BHB9 strain.

### Whole genome sequencing of BHBT5

In order to identify specific mutations responsible for 1-butanol tolerance, the genome of the BHBT5 strain was sequenced via the Illumina Miseq platform. The sequencing achieved a 300× average depth and a >99 % breadth coverage of the reference genome. The breseq results of BHBT5 were compared with that of the parent strain from Mary Lidstrom’s lab in order to identify unique genetic variations in BHBT5 (Tables 1, 2). The complete sequencing results were summarized in Additional file 1. One mutation was identified in the genome of the BHBT5 strain and confirmed by diagnostic PCR, which was a SNP (an A–C transition (L171R)) in the potassium (K^+^)/proton antiporter-coding region (*kefB*) (Tables 1, 2). Only two unique IS elements were discovered in BHBT5 and both of them occurred at the same region (META1_0826) coding for a protein with unknown function.

**Table 1:** Summary of whole genome sequencing of strain BHBT5

Strain	Total number of paired-end reads	Genome coverage rate	Average coverage
BHBT5	9,517,254	99.5 %	324 ± 69

**Table 2:** Unique SNP occurred only in strain BHBT5

Gene ID	Position	Mutation	Gene product
META1_2712	2838079	A→C	Potassium: proton antiporter

### Evolved *kefB* increased 1-butanol tolerance in the wild-type background

To identify the effect of this mutation on 1-butanol tolerance, we introduced the evolved allele in the wild-type background of *M. extorquens* AM1 and compared its growth rate with the BHBT5 strain and wild-type strain in the hypho methanol medium containing 0.15 % (v/v) or 0.5 % (v/v) 1-butanol. The *kefB* mutant was able to grow with a 24.0 % reduced growth rate compared with BHBT5 in the presence of 0.5 % butanol. Furthermore, the *kefB* mutant was more tolerant to 0.15 % 1-butanol than the wild-type strain (Table 3).

**Table 3:** Effect of mutation to *kefB* on 1-butanol tolerance in *M.extorquens* AM1

Strain	Growth rate on methanol (h^−1^)	
	With 0.15 % 1-butanol	With 0.5 % 1-butanol
Wild-type	0.074 ± 0.007	No growth
BHBT5	0.097 ± 0.008	0.075 ± 0.007
Mutation to *kefB*	0.105 ± 0.013	0.057 ± 0.004

### Global metabolomic comparison of BHBT5 and wild-type

**Fig. 5:**
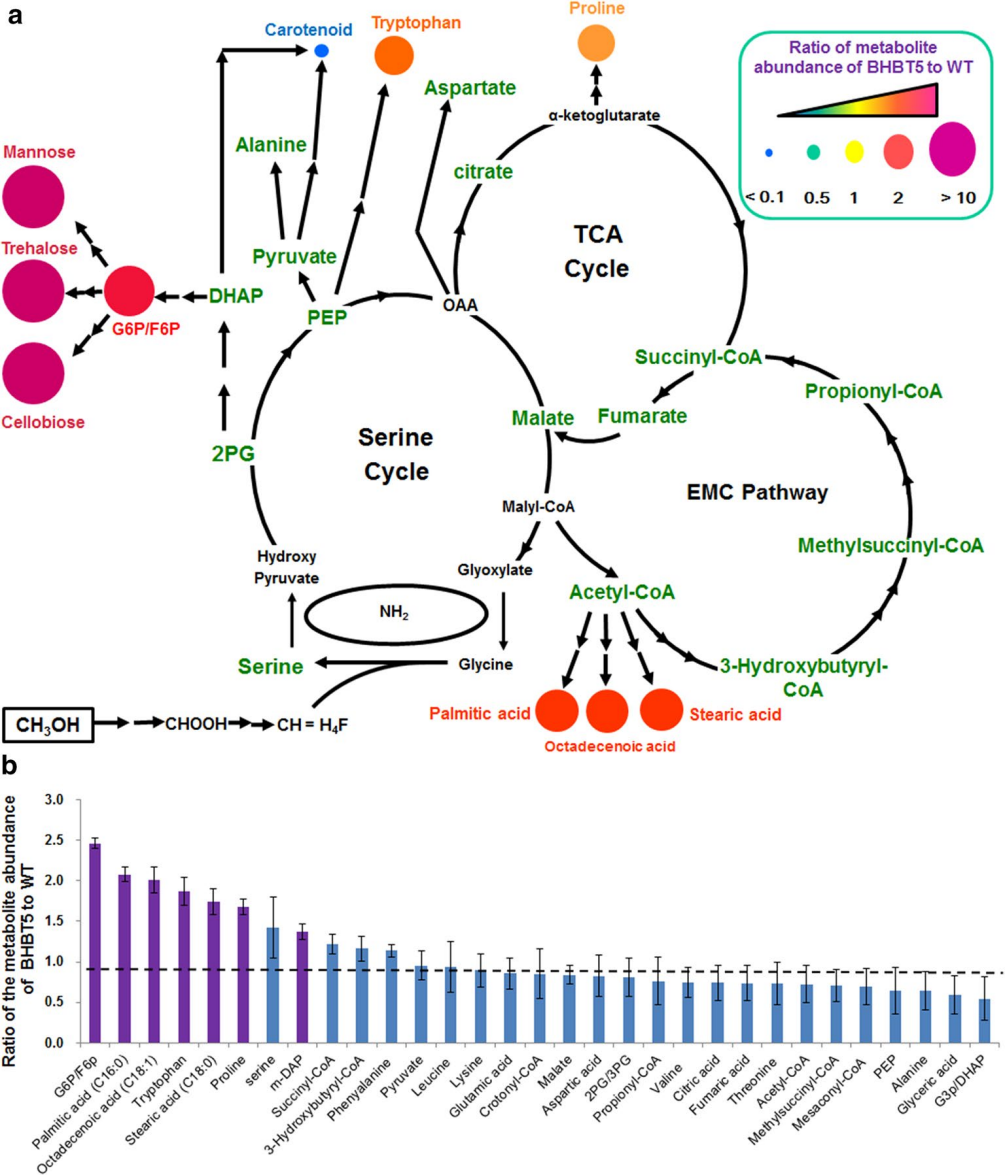
Targeted metabolomic analysis of wild-type and BHBT5 strains. **a** Methylotrophic major metabolism in *M. extorquens* AM1 and metabolite change between BHBT5 and wild-type. The *colored circle* indicates significant difference (*p* < 0.05). The metabolites without significant change are shown in *green*. *Double arrows* indicate multiple reactions. **b** The ratio of metabolite abundance of the BHBT5 strain to the wild-type strain. *Note*: myristic acid (C14:0) was not presented as it was only detected in the wild-type strain. Ratios outside the 1.0 ± 0.2 range indicated significant difference of concentration (*p* < 0.05) for each metabolite (*purple*). The data were presented as the mean plus STDEV calculated from triplicate biological replicates

Targeted metabolite profiling was applied to investigate key metabolites involved in the central C1 and C2 assimilation pathways in *M. extorquens* AM1 (Fig. 5a). There was no obvious disparity between the wild-type and BHBT5 strains in the absence of 1-butanol (Data not shown). However, significant changes were observed among some of the 33 targeted metabolites measured in wild-type and BHBT5 strains incubated in the presence of 0 and 0.5 % of 1-butanol, respectively (Fig. 5b). Carboxylic acids, CoA derivatives and the majority of amino acids in the BHBT5 strain remain similar to that in the wild-type strain, with the exceptions of tryptophan and proline, which showed 1.7-fold and 1.9-fold increases, respectively. Several fatty acids including palmitic acid (C16:0), octadecenoid acid (C18:1), and stearic acid (C18:0) increased (ratio >1.5) in the BHBT5 strain with the supplement of 0.5 % 1-butanol. In contrast, myristic acid (C14:0) was not detected in the BHBT5 strain but abundant in the wild-type strain. Furthermore, Meso-2, 6-diaminopimelic acid (m-DAP), an important precursor intermediate for synthesizing the peptide chain of peptidoglycan in Gram-negative bacteria, was 1.4-fold higher in strain BHBT5. Two phosphate metabolites, G6P and F6P, exhibited a 2.5-fold increase in strain BHBT5, while no significant difference was observed in other phosphate metabolites including 2/3PG, G3P/DHAP, and PEP.

**Fig. 6:**
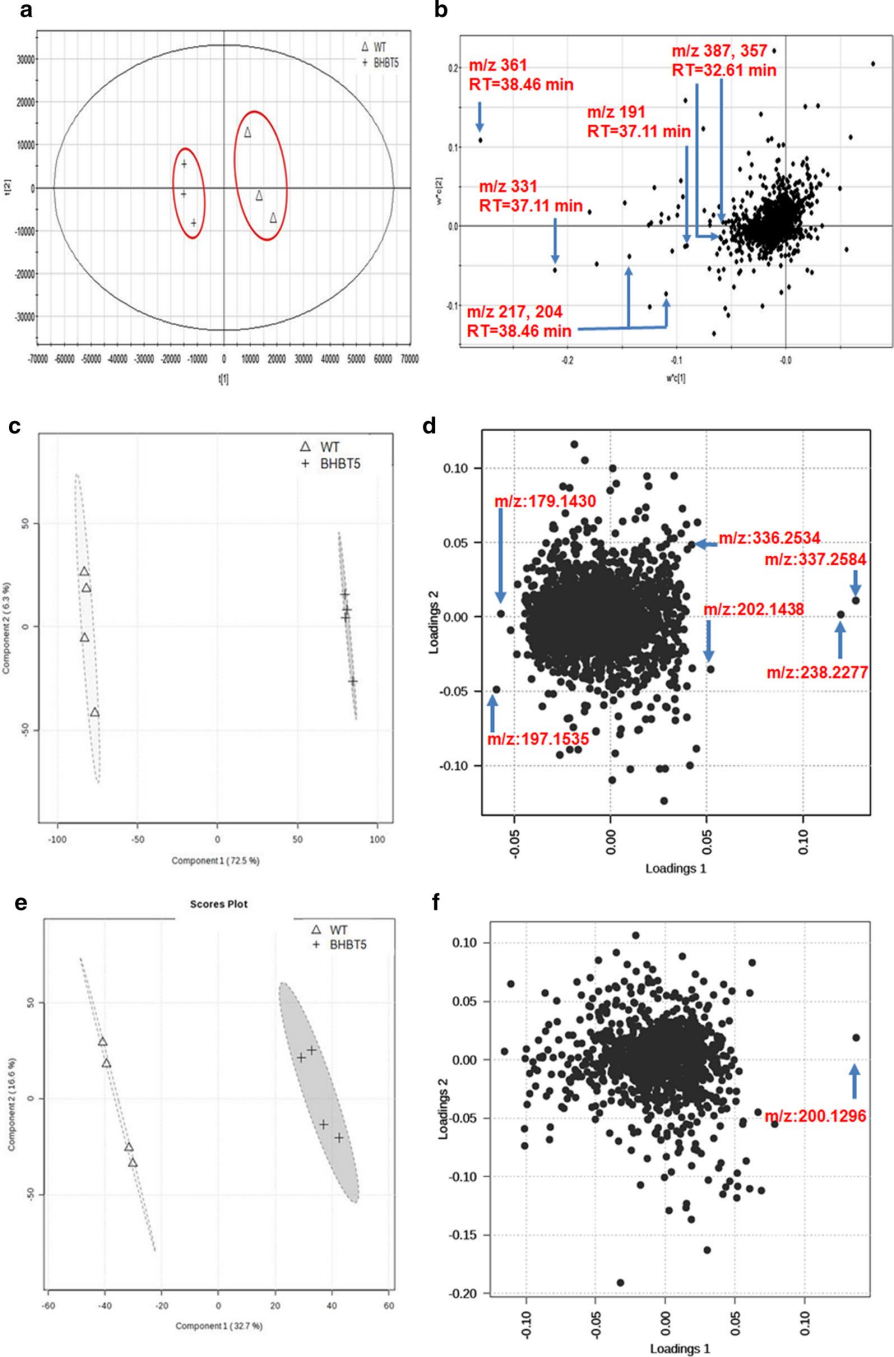
Untargeted metabolomic analysis of wild-type and BHBT5 strains. The metabolome data were processed by PLS-DA. **a**, **b** The score plot and loading plot of metabolome analyzed by GC–MS. **c**, **d** The score plot and loading plot of metabolome analyzed by LC–MS in the positive mode. **e**, **f** The score plot and loading plot of metabolome analyzed by LC–MS in the negative mode. The *arrows* point out the differential metabolites between the wild-type and BHBT5 strains

**Fig. 7:**
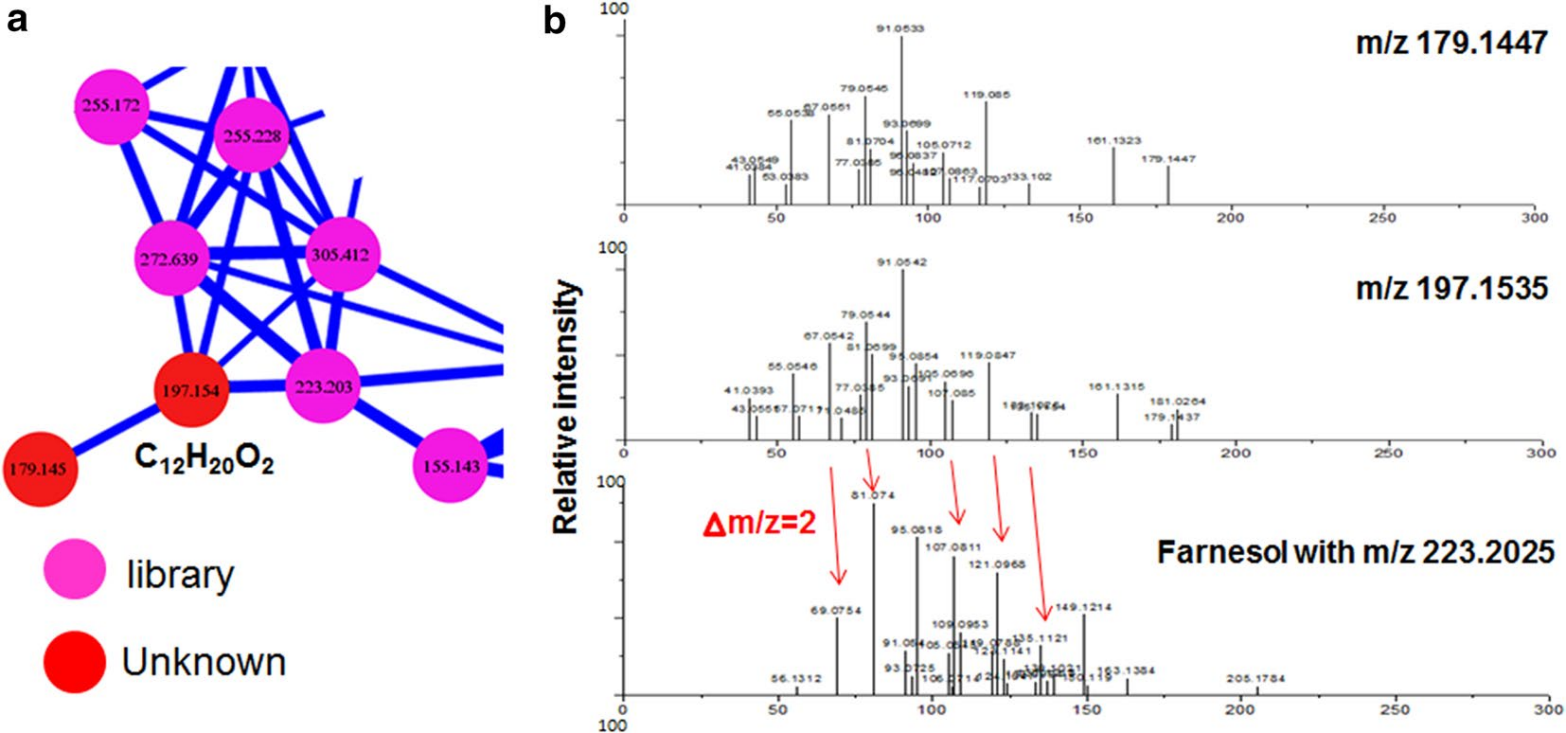
Mass spectral molecular networking was used for comparison of unknown metabolites with the library database. One unknown compound with m/z 197.1535 (m/z 179.1447 is the loss of H_2_O of 197.1535) was clustered with several other compounds. Manual database search revealed that one member of this network was farnesol with m/z 223.2025, which is a precursor of carotenoid synthesis

The untargeted metabolome of the same samples was further analyzed on GC–MS and LC–MS with the assistance of multivariate statistics PLS-DA (Fig. 6). The loading plot revealed that the main variables responsible for the separation between BHBT5 and wild-type strains were nine metabolites (Fig. 6b, d, f). Three of them (i.e., trehalose, cellobiose, mannose) were discovered by GC–MS and identified as sugar compounds against the NIST library (Fig. 6b; Table 3). For LC–MS, six compounds were discovered distinguishable between the BHBT5 and wild-type strains (Fig. 6d, f). MS/MS for each parental m/z was obtained and shown in Fig. 7 and Additional files 2, 3, 4, 5, 6. Molecular networking analysis was further carried out to elucidate the possible structure. Notably, one of six compounds (i.e., elemental composition is C_12_H_20_O_2_), which was detected in the wild-type strain but not in the BHBT5 strain, had a similar MS/MS pattern with farnesol, a precursor of carotenoid synthesis according to the mass spectral molecular networking analysis (Fig. 7; Table 4).

**Table 4:** Differential metabolites were discovered by PLS-DA

GC-MS compound	Match value/elemental composition	m/z	Retention time (min)	Peak abundance	
				Wild-type BHBT5	
Cellobiose	884	361, 217, 204	38.46	NA	1.85E+06
Trehalose	830	331, 191	37.11	NA	3.61E+05
Mannose	910	387, 357	32.61	NA	1.70E+06
LC–MS compound					
Unknown	C_20_H_33_NO_3_	336.2534	2.66	NA	1.38E+06
Unknown	C_16_H_30_N_2_O_3_	299.2335	2.82	NA	8.83E+04
Unknown	C_14_H_27_N_3_	238.2277	14.00	NA	1.40E+05
Unknown	C_10_H_19_NO_3_	202.1438	2.49	NA	2.27E+05
Unknown	C_10_H_19_NO_3_	200.1296	2.53	NA	6.20E+05
Analog of farnesol	C_12_H_20_O_2_	197.1535, 179.1447	3.42	2.57E + 05	NA

The wild-type and BHBT5 strain were grown on methanol with the addition of 0 and 0.5 % 1-butanol, respectively

*NA* not available due to the abundance lower than the limit of detection

## Discussion

*M. extorquens* AM1 has been considered as a potential platform strain for industrial production of valuable chemicals. In previous research, we reported metabolic engineering of the native ethylmalonyl-CoA pathway and heterologous genes in *M. extorquens* AM1 for producing 1-butanol up to 13.6 mg/L [6]. One of the issues limiting the future development of *M. extorquens* AM1 for 1-butanol production is solvent toxicity. In this work, we reported the application of an adaptive laboratory evolution approach to improve 1-butanol tolerance in *M. extorquens* AM1. Based on previous studies, strain fitness during enrichment experiments typically rises rapidly during the first 100–500 generations and slows down considerably during the course of ALE [22]. Therefore, prolonged selection that exceeds the first rapid evolutionary adaptation phase will not necessarily lead to significantly improved phenotypes. This general principle is well applied to the time span of the serial transfer experiments in this study, which were performed for about 200 generations. The evolved BHBT5 strain is able to maintain 50 % of the normal growth rate in 0.5 % (v/v) 1-butanol, a level that can severely damage viability of many industrial microorganisms such as *Pseudomonas putida*, *E.coli* and *Bacillus subtilis* [23]. Overexpression of the 1-butanol synthetic pathway in the BHBT5 strain increased 1-butanol titer by 87 %. Due to its low level, the toxicity of 1-butanol is unlikely to be a key factor limiting 1-butanol production in *M. extorquens* AM1. The increased 1-butanol production therefore is likely a result of altered metabolism in the BHBT5 strain.

Comprehensive molecular analysis of the BHBT5 strain revealed genomic and metabolite variations in response to solvent stresses commonly induced by 1-butanol, such as intracellular ion leakage, reduced intracellular pH, increased membrane fluidity and protein misfolding. Specifically, the genome of the BHBT5 strain was found to contain a SNP [an A–C transition (L171R)] in *kefB,* which codes for a transmembrane potassium (K^+^)/proton antiporter. This mutation was found to provide a significant increase of 1-butanol tolerance in the wild-type background. The identification of *kefB* is very interesting, as recently similar mutations to *kefB* were demonstrated to confer beneficial growth on an engineered *M. extorquens* AM1 strain, which was experimentally evolved to use a GSH-dependent pathway for formaldehyde oxidation [24]. In that case, the parallel SNPs of *kefB* occurred in independent cell lines and were different from our finding. In *E. coli*, *kefB* is a GSH-gated potassium channel that protects cells during electrophilic attack via the modulation of the cellular pH [25]. Current evidence showed that the potassium and pH gradient across the membrane played a role in enhancing alcohol tolerance in *E. coli*, *S. cerevisiae*, and *Clostridium beijerinckii* [26–28]. Since leucine and arginine have quite different hydrophobicities, the L171R mutation may impact the transmembrane function of K^+^/proton antiporter, which in turn may help improve 1-butanol tolerance in *M. extorquens* AM1.

The biosynthesis of long-chain fatty acids such as C18:1 and C18:0 in BHBT5 was increased and the short-chain myristic acid was hardly detectable, suggesting that a denser membrane packing might contribute to alcohol tolerance in the BHBT5 strain grown with 1-butanol. In *M. extorquens* AM1, the intermediate G6P is generated through a reverse glycolysis pathway, which is mainly used to synthesize glycogen or constituents of cell wall. M-DAP is a unique intermediate for synthesizing peptide chain of peptidoglycan. Thus, increased G6P and m-DAP in the BHBT5 strain could play a part in peptidoglycan synthesis for cell wall enhancement.

Two amino acids, proline and tryptophan, increase significantly in the BHBT5 strain. Intracellular levels of proline and tryptophan are associated with ethanol tolerance in *S. cerevisiae.* Mutants defective in genes involved in biosynthesis of proline and tryptophan were more sensitive to ethanol stress [29]. Additionally, accumulation of both amino acids was observed in strains with increased ethanol tolerance [30, 31]. The contribution of proline to ethanol tolerance was ascribed to its role in the enhancement of protein stability and prevention of protein aggregation under solvent challenge [32], while the mechanism involving tryptophan remains unknown.

Moreover, metabolomic discovery analysis identified three disaccharides highly upregulated in the BHBT5 strain, but barely detected in the wild-type, including trehalose, mannose, and cellobiose. Although none of the disaccharide biosynthesis pathways has been investigated in *M. extorquens* AM1 so far, homologs of genes related to the corresponding disaccharide production have been identified in the genome of *M. extorquens* AM1 (Trehalose: META1_1441, META1_4841, META1_3486 and META1_3093; Mannose: META1_5250; Cellobiose: META1_1169). Trehalose is a non-reducing disaccharide consisting of two glucose monomers (α-d-glucopyranosyl-1, 1-α-d-glucopyranoside). Because trehalose is relatively inert and very stable, it was reported to be able to reduce membrane permeability as well as ensure proper folding of proteins [33]. In *S. cerevisiae*, trehalose accumulation was observed under ethanol stress, and cells unable to accumulate trehalose displayed retarded growth under ethanol challenges [34]. The functions of mannose and cellobiose in solvent tolerance are not clear. However, the increase of G6P in the BHBT5 strain may stimulate disaccharides synthesis, as G6P is an important precursor for all three disaccharides.

One interesting phenomenon is that the BHBT5 strain produces much less pink carotenoid than the wild-type strain, which makes the BHBT5 strain appear colorless. Furthermore, metabolome data discovered that an analog of farnesol, a precursor of carotenoids synthesis, was strongly reduced in the BHBT5 strain. Although carotenoids are hydrophobic compounds that affect the membrane physical properties such as membrane fluidity and permeability of small molecules [35], their impacts on solvent tolerance have not been reported. The carotenoid synthetic pathway in *M. extorquens* AM1 consists of the 1-deoxy-d-xylulose-5-phosphate (DXP) pathway for isopentenyl pyrophosphate (IPP) synthesis and a similar hydroxyspheroidene pathway to *R. sphaeroides* for conversion of IPP to a mixture of bacterioruberin- or oscillaxanthin-like carotenoids [36]. Genome analysis and diagnostic PCR show that none of the mutations occurred at the genes involved in the carotenoid synthetic pathway (data not shown). So further information is required to understand the relationship between carotenoid production and 1-butanol tolerance in the BHBT5 strain. Notably, a previous study demonstrated that a *M. extorquens* AM1 mutant strain deficient in the ethanol response regulator *phyR* was less pigmented than the wild-type and less tolerant to 2 % ethanol [11]. This contradiction suggests that *M. extorquens* AM1 may evolve separate mechanisms in response to the toxicity of long-chain and short-chain alcohol as shown in other bacteria [16, 17].

In addition, metabolome analysis discovered several unknown metabolites that significantly accumulated in the BHBT5 strain. Additional work needs to be conducted to understand how these changes contribute to 1-butanol tolerance in *M. extorquens* AM1.

## Conclusion

As a next step towards development of *M. extorquens* AM1 as an industrial platform, adaptive laboratory evolution was used as a tool in this work to develop *M. extorquens* AM1 for high 1-butanol tolerance. We applied a serial transfer method to isolate two mutant strains, BHBT3 and BHBT5, both of which demonstrated increased fitness compared to the parent strain in the presence of 1-butanol. Strain BHBT5 exhibited increased 1-butanol production from 13.6 to 25.5 mg/L after introducing the 1-butanol synthetic pathway. Whole genome sequencing of the BHBT5 strain identified that a point mutation at *kefB* may play a crucial role in 1-butanol tolerance. Global metabolomic analysis revealed that several key diagnostic metabolites including trehalose and tryptophan were significantly upregulated in response to the 1-butanol stress in the BHBT5 strain. On the contrary, the carotenoid synthesis pathway was strongly down-regulated in the mutant strain. The genes involved in those metabolite pathways can be harnessed in attempts to increase the tolerance and rebalance 1-butanol production and cell growth in the future. The collected information from this research will be useful for uncovering the mechanism of cellular response of *M. extorquens* AM1 to solvent stress, and will provide the genetic blueprint for the rational design of a strain of *M. extorquens* AM1 with increased 1-butanol-tolerance in the future.

## Methods

### Strain, medium, and growth condition

*Escherichia coli* strains Top 10 and S17-1 were cultivated at 37 °C in Luria–Bertani medium. *M. extorquens* AM1 wild-type and 1-butanol-tolerant mutants were cultured in a minimal medium (hypho) supplemented with 1.77 µg/L CoCl_2_ [37]. One of the following substrates was used as carbon source: succinate (20 mM), methanol (125 mM), or ethylamine (20 mM). Triparental matings between *E. coli* and *M. extorquens* AM1 were conducted on Difco nutrient agar plate. Antibiotics were supplied at concentrations as follows: kanamycin (Km), 50 μg/mL, and rifamycin (Rf), 50 μg/mL.

### Adaptive evolution of *M. extorquens* AM1

A sequential transfer method was used to isolate 1-butanol-tolerant mutants of *M. extorquens* AM1. *M. extorquens* AM1 was first streaked on a hypho methanol plate and a single colony was picked to inoculate 5 mL of liquid hypho methanol culture, which was grown to the OD of 1.0 (OD_600_) as a preculture. The preculture was then diluted into twenty 25 mL plastic-cap tubes (1:100) containing 5 mL of hypho methanol medium and 0.15 % 1-butanol (v/v). Cultures were inoculated at 30 °C for 72 h to late exponential phase and the culture of highest OD_600_ was selected as the seed culture for the next round of transfer. A series of transfers were conducted at the same concentration of 1-butanol (3–5 transfers). Then cultures were diluted and spread on hypho methanol agar plates containing 0.15 % 1-butanol to pick single colonies for the next round of inoculation, in which the 1-butanol concentration in the medium was increased to 0.2 %. The transfer process was continuously repeated with incremental 1-butanol concentration of 0.05 % each time to the final concentration of 0.5 %. Mutants with improved 1-butanol tolerance were stored in 10 % DMSO at −80 °C.

### 1-Butanol tolerance of selected mutants

Cultures of *M. extorquens* AM1 wild-type and two 1-butanol-tolerant mutants (BHBT3 and BHBT5) were inoculated to middle exponential phase in 5 ml hypho methanol medium at 30 °C, 220 rpm. Then 0.5 ml of culture at OD of 1.0 was distributed into 50 ml fresh hypho methanol medium in 250-ml flasks containing appropriate amounts of 1-butanol. Flasks were incubated at 30 °C with shaking at 220 rpm. OD_600_ of the growing cultures was measured every 3 h until stationary phase. The specific growth rates of cultures were calculated from the log-linear growth phase using Microsoft Excel^®^. The growth rates presented for each strain and condition are the mean plus STDEV calculated from triplicate biological replicates. For viable cell counting, cultures inoculated after 24 h were diluted with fresh hypho medium and spread on hypho methanol plates. Colonies formed after 3 days of incubation were counted (50–500 per plate). Triplicate experiments were performed for each individual condition.

### Survival rates of *M. extorquens* AM1 under high 1-butanol pressure

Survival rates of mutated strains were assessed using the method described previously [38]. Single colonies of the mutated strain were inoculated in plastic tubes and subcultured into 250-mL flasks containing 50 mL of hypho methanol medium. After 24 h of incubation, 0.5 mL of mid-log phase culture was mixed with 4.5 mL hypho methanol medium supplemented with 0.25 g 1-butanol (final 1-butanol concentration 50 g/L, i.e., 6.17 % butanol (v/v)). The culture was then mixed and on the benchtop for 30 min, which was followed by serial dilutions using fresh hypho medium. The diluted culture was spread onto hypho methanol plates and incubated at 30 °C for 3 days before colony counting. A control experiment was set up using the same dilution procedure without the addition of 1-butanol. Cell survival rate was calculated as colony forming units (cfu) per mL of butanol-exposed culture divided by that of control culture.

### Genomic DNA extraction and whole genome sequencing

The genomic DNA of BHBT3 and BHBT5 were extracted using a phenol/chloroform extraction protocol as described before [39]. Cells grown on methanol were harvested at mid-exponential phase and resuspended in 5 mL of lysis buffer (10 mM NaCl, 20 mM pH 8.0 Tris–HCl, 1 mM EDTA and 2 % (w/v) SDS). Cell lysates were incubated with 50 µl of 100 mg/mL RNaseA and 250 µL of proteinase K overnight at 55 °C. The DNA was separated from protein and RNA by phenol/chloroform extraction and recovered via ethanol precipitation. The DNA samples for whole genome sequencing were dissolved in 400 µL of TE buffer. Preparation of paired-end libraries and whole-genome sequencing were performed by Genewiz Inc. (Plainfield, NJ) using the Illumina-Miseq sequencing platform.

To detect changes between the sequenced strain and the butanol tolerant strains, the results of three independent analyses were combined. First, the raw reads were aligned to the genome and processed using *breseq* version 0.19 [40]. Next, the raw reads were quality filtered using *Nesoni* version 0.122 (Nesoni 2014). The filtered reads were assembled using *SPAdes* version 3.0 [41]. The assembled contigs were compared to the published sequence using in-house scripts based on processing the BLAST version 2.2.10 [42]. Finally, the filtered reads were aligned to the published scaffold with BWA version 0.7.5a-r405 [43] and the results were post-processed with SAMtools version 0.1.19-44428 cd [44] for variant calling. All predicted mutations were further confirmed by diagnostic PCR with primers listed in Additional file 7.

#### Strain construction

A plasmid (pBH19)-containing genes coding for the 1-butanol synthetic pathway in strain BHB9 as reported in a previously published paper was introduced into the strain BHBT5 by bacterial conjugation [6]. 1-butanol production of the new strain, designated BHB10, was evaluated in 50 mL of hypho methanol medium inoculated at 30 °C, 220 rpm.

Allelic exchange was performed using modified pCM433, a *sacB*-based suicide vector, in which the tetracycline resistance marker was replaced by kanamycin [45, 46]. Briefly, a PCR product of the evolved allele (*kefB,* META1_2712) from the BHBT5 was inserted into pCM433 using primers p2712-NdeI-Fw and p2712-SacI-Rev to generate pJY25. The primers were listed in Additional file 7. The plasmid was then introduced into the wild-type *M. extorquens* AM1 using triparental mating with the helper plasmid pRK2103 [47]. Single-crossover mutants were selected using kanamycin resistance, and double-crossover mutants selected by growth on plates containing 5 % w/v sucrose. Successful allele swapping was confirmed by diagnostic PCR with gene sequencing.

### Global metabolomic analysis

Samples (10 mL) of mid-exponential cultures were rapidly harvested by vacuum filtration using MILLEX-GP PES membrane filters (0.22 µm, 33 mm) (Millipore; Billerica, MA, USA) as described before [48]. Extraction of metabolites was carried out as previously published for *M. extorquens* AM1 with slight modification [49]. Briefly, the samples with internal standards were incubated in a 100 °C water bath for 3 min. The extracted cell suspension was cooled on ice for 5 min, then cell debris was removed by centrifugation at 5000 rpm for 5 min. The cell-free metabolite extract was centrifuged at 14,000 rpm for 8 min. The supernatant was dried in a rotational vacuum concentrator (Christ Gefriertrocknungsanlagen Gmbh, Germany) and stored at −80 °C. For LC–MS analysis, each dried sample was dissolved in 50 mL of purified water. For GC–MS analysis, each sample was further derivatized in two steps. First, keto group were methoximated by adding 50 mL of methoxyamine solution (25 mg/mL methoxyamine hydrochloride in pyridine) and incubated at 60 °C for 30 min. Second, trimethylsilylation was performed by adding 50 mL of a TMS reagent (BSTFA/TMCS, 99:1) and incubated at 30 °C for 90 min. For fatty acids analysis, whole cell hydrolysis with subsequent acid methylation of fatty acids was carried out as described [50] with slight modifications. Cells (18 mg CDW) were hydrolyzed with 4 mL of 15 % NaOH (w/v) in methanol/water (1:1, v/v) for 30 min at 100 °C. Fatty acid methyl esters (FAMEs) were obtained by adding 8 mL of 6 M HCl/methanol (13:11, v/v) and incubation for 2.5 h at 80 °C. FAMEs were extracted with 5 mL of hexane/methyl-tertbutyl ether (1:1, v/v) and washed with 6 mL of 1 % NaOH in water (w/v).

LC–MS experiments were carried out on either an Agilent LC-QQQ-MS system (Agilent 1290 Infinity-6460, Agilent Corp, SantaClara, CA, USA) or Agilent LC-QTOF (Agilent 1290 Infinity-6530B, Agilent Corp, SantaClara, CA, USA). For LC-QQQ-MS, multiple reaction monitoring (MRM) precursor/product ion pairs were carried out as before [51]. For LC-QTOF, the m/z range was set to 50–1200 in centroid mode with a scan rate of 1.5 spectra/s. The ESI conditions were as follows: capillary voltage of 4000 V, fragmentor of 135 V, gas temperature of 300 °C, nebulizer of 35 psig, gas flow of 10 L/min. For internal calibration, G1969-85001 ES-TOF Reference Mass Solution Kit was used and reference nebulizer was set at 3 psig. The LC method was carried out using Waters Acquity UPLC BEH Amide column (150 × 2.1 mm, 1.7 μm). Mobile phase A consisted of 0.1 % (V/V) formic acid and 0.075 % (v/v) ammonium hydroxide (28 %) in water/acetonitrile (2:98, v/v), while mobile phase B was acetonitrile with water (95:5, V/V). The linear gradients used were 100 % B for 4 min, 100–60 % B for 17 min, 60–25 % B for 4 min, 25–100 % B for 1 min, and 100 % B for 13 min. The flow rate was 0.2 mL/min and the column was set at 30 °C. GC–MS experiments were performed using an Agilent 5975B/6890 N GC–MS (Agilent Corp; SantaClara, CA, USA). The column was HP-5MS (30 m × 0.32 mm × 0.25 mm film; Restek, Bellefonte, PA, USA). 1 μL of a given sample was injected in split-less mode through an Agilent7693 autosampler. The inlet temperature was set to 280 °C. The temperature began at 60 °C and then increased at 5 °C/min to 280 °C where it was held for 10 min. The ion source temperature was set to 240 °C. The targeted metabolomic analysis was carried out as described before [51]. The peaks were analyzed using Agilent ChemStation software. For the untargeted metabolome analysis, LC–MS and GC–MS data were converted into mzML format using MS Convert software. Data preprocessing and statistical analysis were performed with MZmine 2.10 and Metaboanalyst 3.0 or SIMCA-P v11.5 [52, 53]. For molecular network analysis, the MS/MS data were converted to mzXML and then were processed using a web-based server as previously described [54]. The molecular networking data were then visualized using Cytoscape 2.8.2.

### 1-Butanol and carotenoid measurement

1-butanol production of BHB10 was determined by GC–MS with the method described before [6]. Briefly, ten mL of culture samples was centrifuged for 10 min at 5000 rpm. Two mL of ethyl acetate was added to the supernatant. The recovered ethyl acetate was analyzed by a HP 6890 gas chromatograph equipped with a Model 19091 s-433 HP-5MS column (Agilent) and an Agilent 5973 single quadrupole mass spectrometer. The peaks were analyzed using Agilent ChemStation software. Carotenoids were extracted from *M. extorquens* AM1 as previously described [36]. Briefly, cell pellets obtained from 100 mL of cell culture were resuspended in 1 mL of methanol at 65 °C, followed by the addition of 0.4 mL of water and 0.3 mL of chloroform, then vortexed. The bottom organic layer containing the carotenoids was transferred into clean tubes. Then 1 mL of methanol and 0.4 mL of water were added and vortexed and the organic layer was transferred again. The sample was placed at −20 °C overnight and then was centrifuged. The supernatant was evaporated to dryness and redissolved in 0.1 mL of chloroform for a Waters HPLC 1260 analysis. The separation on HPLC was conducted with a Waters Acquity BEH C18 column (100 × 2.1 mm, 1.7 μm). The mobile phase consisted of methanol–MTBE–water (solvent A, 85:14:5, v/v/v) and methanol–MTBE–water (solvent B, 90:5:5, v/v/v). The gradient program was set as follows: 0 % A to 20 % in 3 min, 20 % A to 100 % in 6 min, and retained from 6 to 20 min. The flow rate was 0.3 mL/min and the UV absorbance of the peaks was collected from 200 to 620 nm using a photodiode array detector and monitored at 460 nm. The beta-carotene was used as a standard to confirm the identification.

## Additional files

10.1186/s13068-016-0497-y The complete genome sequencing results of BHBT5 and the parent strain from Mary Lidstrom’s lab.
10.1186/s13068-016-0497-y MS/MS of m/z 200.1292.
10.1186/s13068-016-0497-y MS/MS of m/z 202.1438.
10.1186/s13068-016-0497-y MS/MS of m/z 238.227.
10.1186/s13068-016-0497-y MS/MS of 336.253.
10.1186/s13068-016-0497-y MS/MS of m/z 299.2335.
10.1186/s13068-016-0497-y The primers used in this study.

## Abbreviations

ALE: adaptive laboratory evolution
m-DAP: meso-2, 6-diaminopimelic acid
G6P: glucose-6-phosphate
F6P: fructose-6-phosphate
2/3PG: 2/3-phosphoglycerate
G3P/DHAP: glyceraldehyde-3-phosphate/dihydroxyacetone phosphate
PEP: phosphoenolpyruvate
CFU: colony forming unit
GSH: glutathione
