# Supplementary figures and images for: Comprehensive molecular characterization of Methylobacterium extorquens AM1 adapted for 1-butanol tolerance
Source: Biotechnol Biofuels. 2016 Apr 11;9:84. doi: 10.1186/s13068-016-0497-y (PMC4827201; doi:10.1186/s13068-016-0497-y)

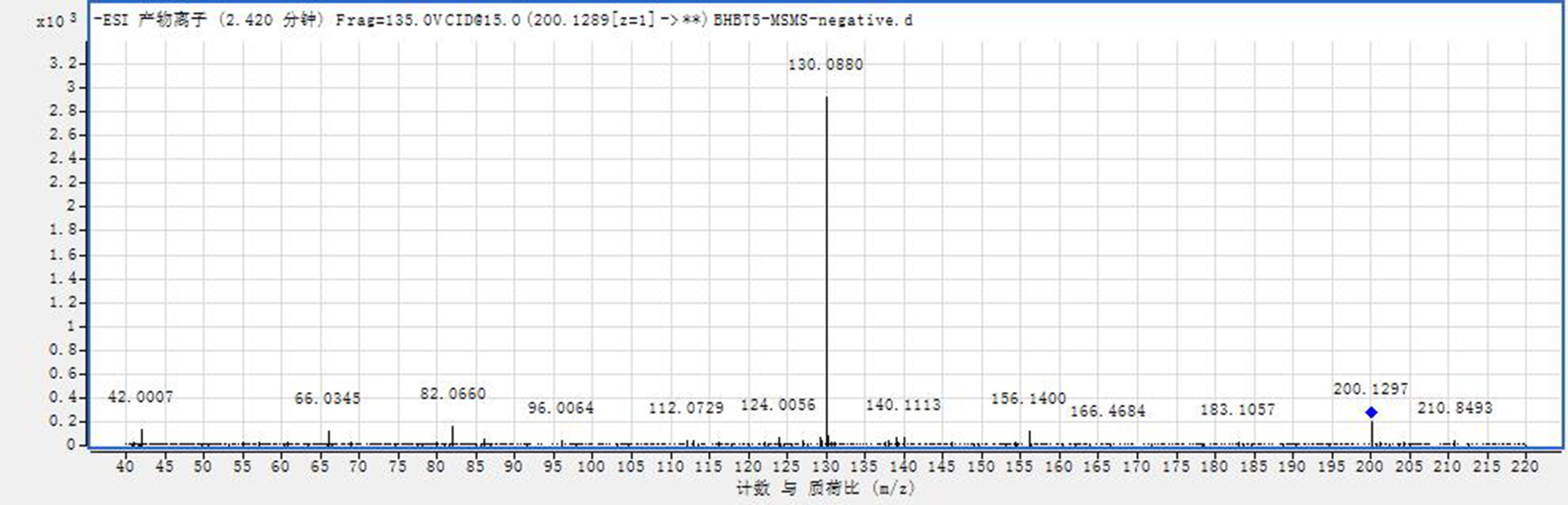

Supplement: Supplementary file 2 — 10.1186/s13068-016-0497-y MS/MS of m/z 200.1292. [file 13068_2016_497_MOESM2_ESM.tif]

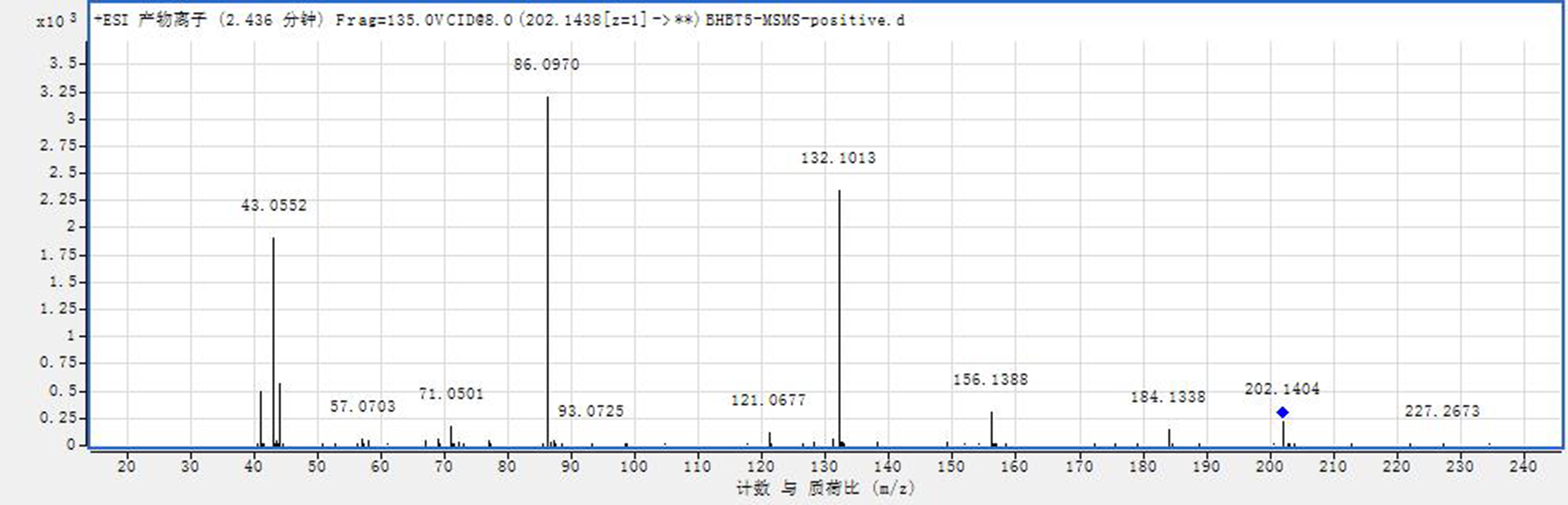

Supplement: Supplementary file 3 — 10.1186/s13068-016-0497-y MS/MS of m/z 202.1438. [file 13068_2016_497_MOESM3_ESM.tif]

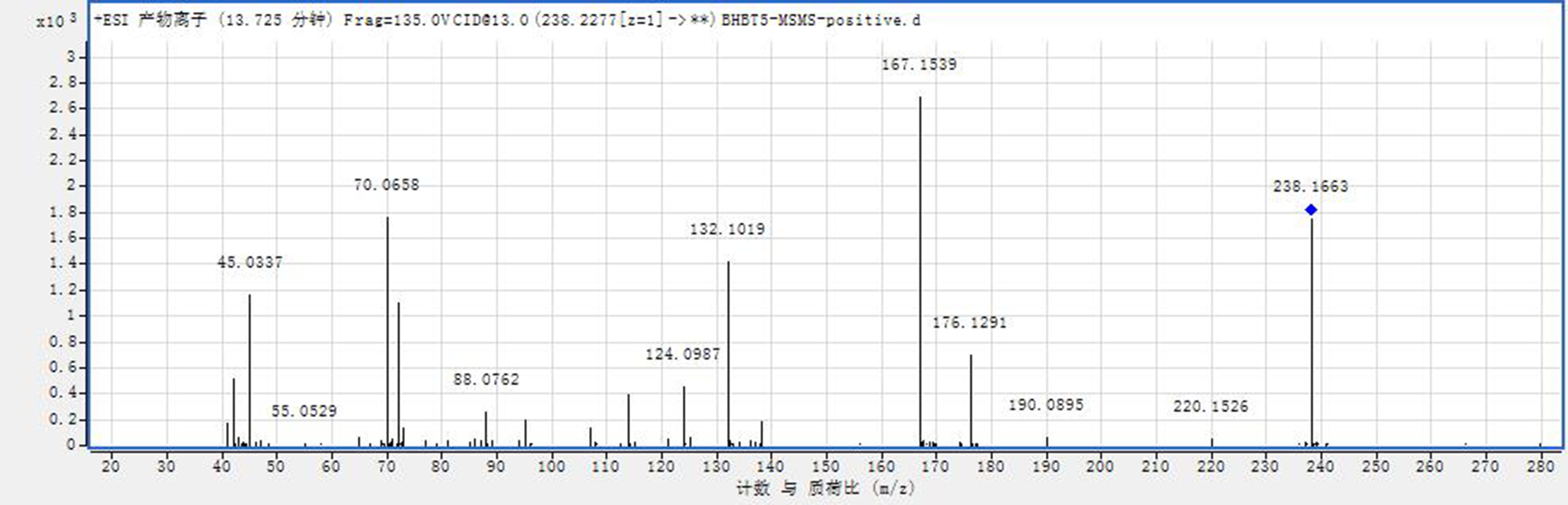

Supplement: Supplementary file 4 — 10.1186/s13068-016-0497-y MS/MS of m/z 238.227. [file 13068_2016_497_MOESM4_ESM.tif]

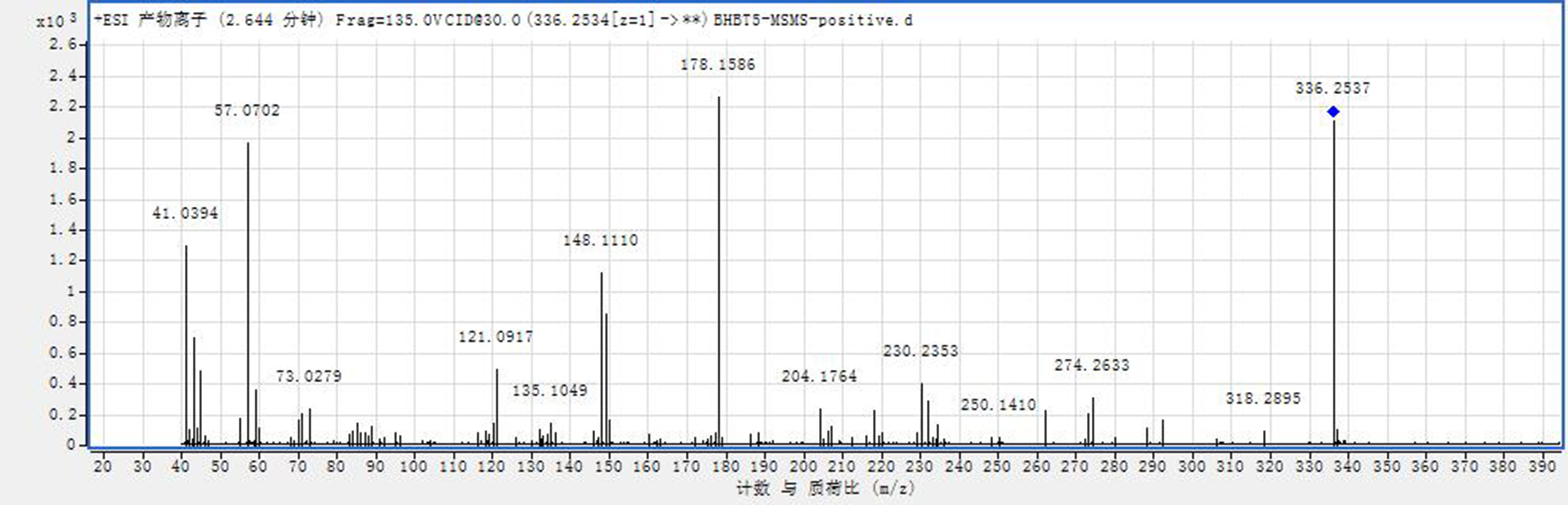

Supplement: Supplementary file 5 — 10.1186/s13068-016-0497-y MS/MS of 336.253. [file 13068_2016_497_MOESM5_ESM.tif]

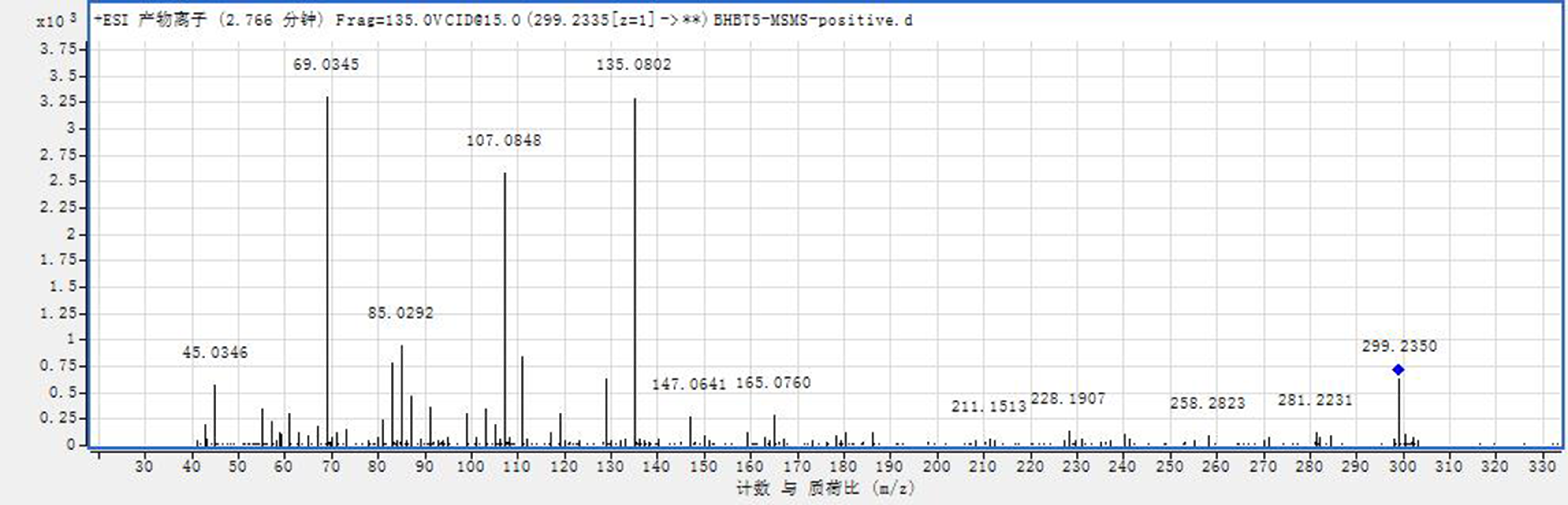

Supplement: Supplementary file 6 — 10.1186/s13068-016-0497-y MS/MS of m/z 299.2335. [file 13068_2016_497_MOESM6_ESM.tif]
